# Twin Reversed Arterial Perfusion Sequence: Assessing the Role of the Correct Imaging Modality in a Rare Clinical Entity

**DOI:** 10.7759/cureus.2910

**Published:** 2018-07-02

**Authors:** Imrana Masroor, Sarah Jeelani, Aliya Aziz, Romana Idrees

**Affiliations:** 1 Department of Radiology, Aga Khan university Hospital Karachi , Karachi, PAK; 2 Post Graduate Medical Education, Aga Khan University Hospital, Karachi, PAK; 3 Department of Obstetric and Gynecology, Aga Khan University Hospital, Karachi, PAK; 4 Department of Pathology, Aga Khan University Hospital, Karachi, PAK

**Keywords:** twin reversed arterial perfusion syndrome, prenatal ultrasonography, placental abnormality, pregnancy complications, fetal disease, umbilical artery doppler

## Abstract

Acardiac twin formation is a rare anomaly. It is one of the most extreme complications of monozygotic twin pregnancies. Such occurrences are brought about when a normal twin donates blood to an abnormal twin through its umbilical arteries via vascular anastomoses at the level of the placenta, which is termed as twin reversed arterial perfusion sequence (TRAPS). Twin reversed arterial perfusion sequence is considered a rare variant of twin-to-twin transfusion syndrome. Due to the considerable blood transfer from the healthy twin to the parasitic one, cardiac failure can ensue in the healthy twin. The mortality of the acardiac twin is 100%. We present an obstetric case of a South Asian female, whose serial ultrasound scans consistently displayed a heterogeneous mass, initially labeled a teratoma. This was postoperatively diagnosed as an acardiac twin due to TRAPS. Thus, we would like to highlight the importance of umbilical artery Doppler in the prompt diagnosis of TRAPS so timely management may be undertaken to prevent morbidity and/or mortality of the normal twin.

## Introduction

Acardiac twin formation is a rare anomaly occurring in 1% of monochorionic pregnancies [1]. It is one of the extreme complications of monochorionic monozygotic twin pregnancies; however, it has the potential of being present in all pregnancies of higher multiples. This occurs due to a normal twin donating blood to an abnormal one, which either lacks a fetal heart or possesses a severely malformed one. This transfer of blood occurs through umbilical vascular anastomosis between the normal and acardiac twins, leading to a reversal of perfusion in the acardiac twin, and is thus termed twin reversed arterial perfusion sequence (TRAPS). The morphology of the acardiac twin can vary considerably from a teratoma-like mass to a discernable, yet malformed, fully grown fetus. Here, we present the case of a 34-year-old female who encountered such a condition in our care.

## Case presentation

**Figure 1:**
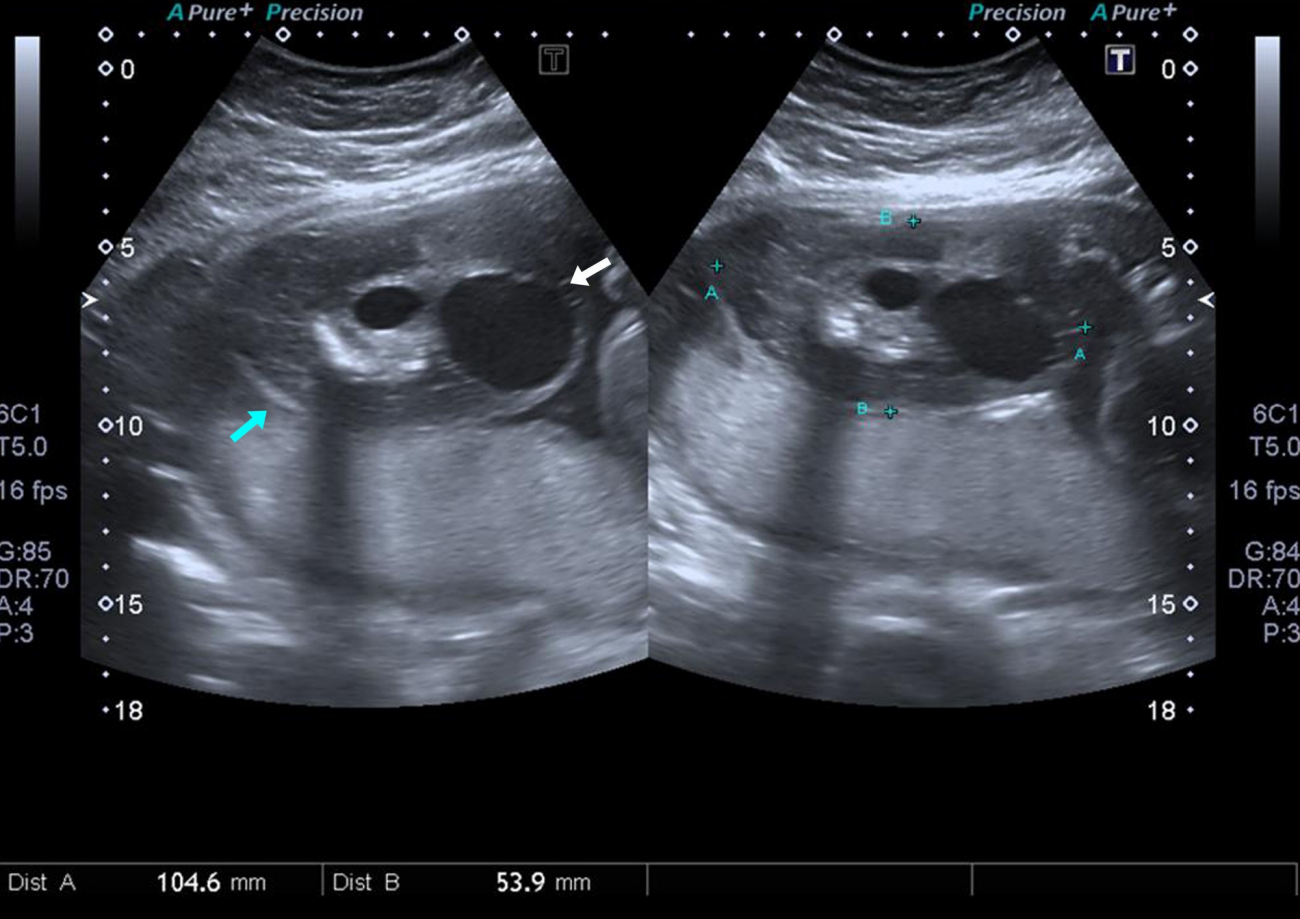
Second-trimester ultrasound scan. A complex multiloculated cystic mass with a larger cystic component (white arrow) and septations and calcifications (blue arrows) can be seen.

A 34-year-old primigravida was referred to our tertiary care hospital with a suspicion of either a chorioangioma of the placenta or a placental teratoma on the second-trimester anomaly scan performed from a secondary care hospital. The patient had no known comorbidities apart from being diagnosed with pregnancy-induced hypertension. The conception was planned and was not assisted by any interventional or medical means. There was no history of congenital anomalies in the family or of twin pregnancies. Ultrasonography at our institution revealed a single intrauterine fetus, corresponding to 27 weeks and two days of gestation, displaying a normal interval growth from the previous ultrasound. The presence of a multiloculated cystic mass with septations and calcifications was also noted. This mass was devoid of any vascularity (Figure 1).

On color Doppler examination, no vascularity was noted and the mass, reported as measuring 104 x 53 mm, appeared as arising from the anterior wall of the uterus. The differential diagnosis of placental teratoma, chorioangioma, hemorrhagic placental degeneration, or degenerating fibroid was considered. A repeat scan in the third trimester revealed the same findings.

**Figure 2:**
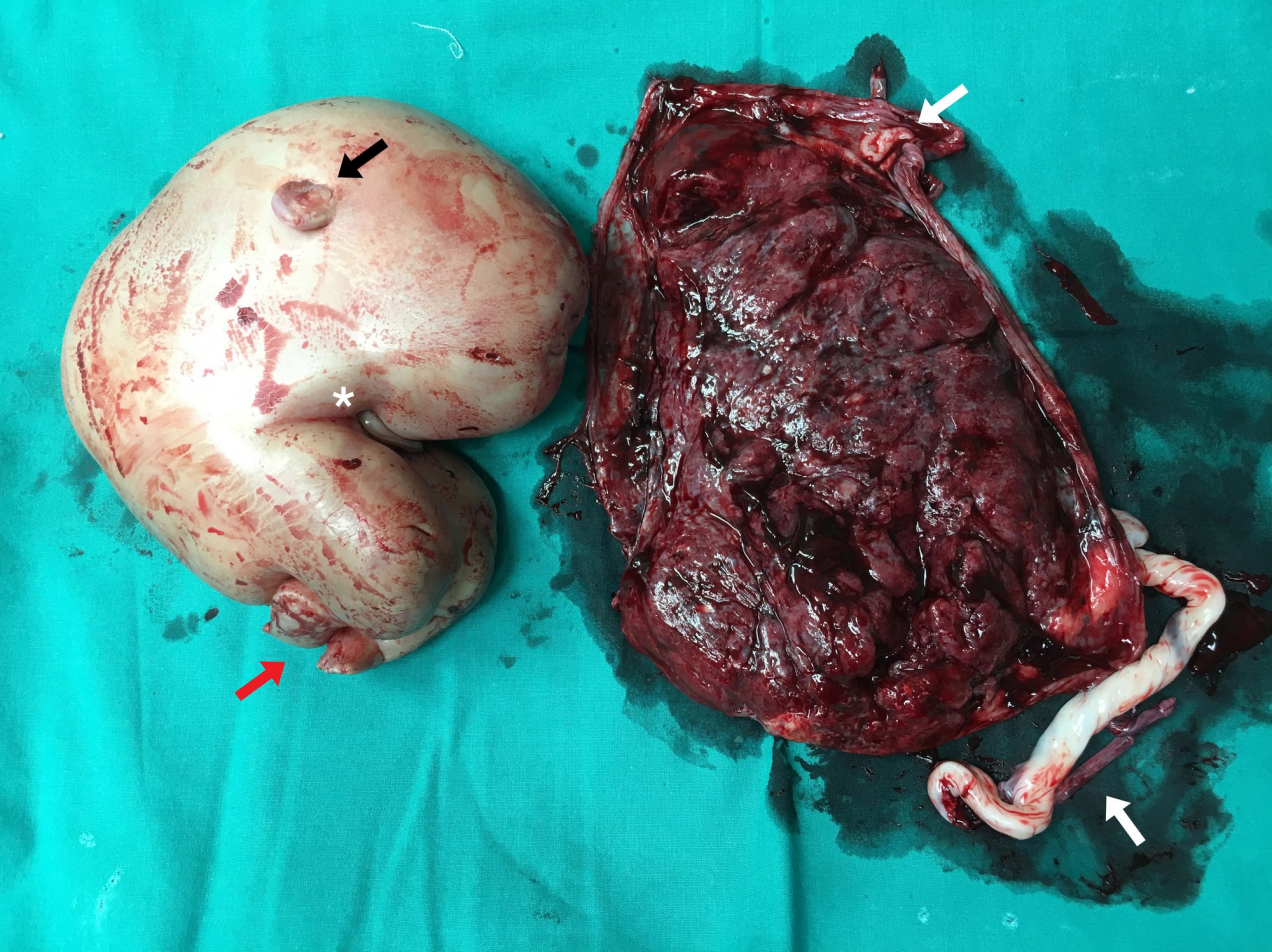
Intraop image of the acardiac twin (left) with placenta (right). Incomplete lower limb formation (red arrow) and partially formed external aurical can be seen (black arrow). Placenta shows the presence of two umbilical cords (white arrows) with concurrent umbilical cord remnant seen in the acardiac twin (white asterisk).

At 36 weeks and five days of gestation, the patient gave birth, via C-section, to a fetus with good Apgar scores (9/1, 9/5). A second congenitally malformed fetus was also delivered, which was devoid of cephalic end structures. Only the feet and lower limbs of the fetus were discernable (Figure 2). A single placenta measuring 515 grams was also delivered, containing two umbilical cords. The patient was discharged in a stable condition on the sixth day of admission.

A histopathological examination of the placenta revealed placental discs measuring up to 19.5 x 14 x 4 cm. The placental membrane measured 30 x 16 cm, with two umbilical cords. The separately lying fetus showed gross malformation with the absence of the head and both upper limbs. However, both lower limbs were partially formed. The heel to rump length was 18 cm. A diagnosis of chorioangiosis of the placental disc along with a malformed twin was made.

**Figure 3:**
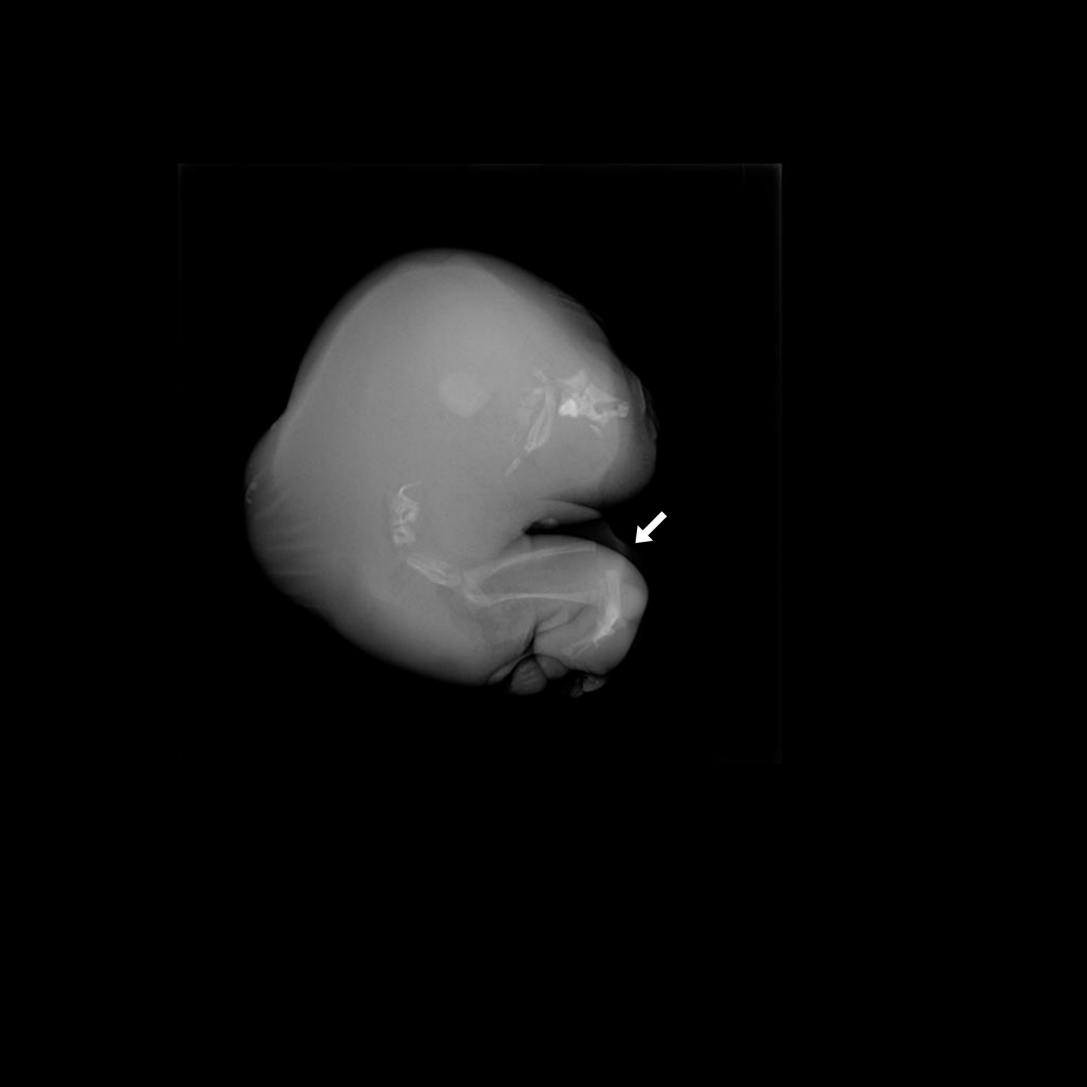
Post-mortem X-ray of the acardiac fetus. Calcification pattern in the lower limbs suggestive of incomplete formation of bilateral femur (white arrow) along with partial formation of sacral spine. Few disorganized calcifications noted in the cranial end.

The post-mortem X-ray ( Figure 3) of the malformed twin showed the incomplete formation of the lower limbs and sacrum with some calcifications present in the cephalic region. This lead to the diagnosis of an acardiac anencephalic twin.

## Discussion

Recent literature reveals that TRAPS occurs in 1:9500 to 1:11000 pregnancies [2]. This phenomenon can be explained by the fact that there are new avenues available for assisted conception, which increases the likelihood of monochorionic twin pregnancies. Complication rates are much higher with monochorionic, monoamniotic twins than those with dichorionic twins. In TRAPS, the normal twin donates blood to the abnormal twin through its umbilical arteries via vascular anastomoses at the placental level. The anomalous twin appears as a heterogeneous mass, simulating a teratoma or even intrauterine fetal demise [3]. Our purpose of reporting this case of TRAPS, which was previously misdiagnosed as a placental tumor, was to highlight the importance of considering a Doppler umbilical artery ultrasound and a gray-scale examination for the confirmed exclusion of TRAPS. This diagnosis becomes crucial, as TRAPS can lead to higher morbidity and mortality for the normal twin and is easily manageable once detected.

Twin reversed arterial perfusion syndrome leads to the most severe form of twin malformations possible and is more common in higher multiple pregnancies. The exact etiology as to why this occurs is based on the presence of two pathological anastomoses early on in gestation. An artery-to-artery anastomosis, which brings blood from the “donor twin” to the acardiac one, and a vein-to-vein anastomosis, which returns this blood, are present. Higher blood pressures in the donor twin cause the flow of deoxygenated blood from its umbilical artery to the acardiac twin’s umbilical artery, which then proceeds in a caudal to cranial direction. Preferential perfusion of the lower limbs can be explained by the close proximity of the legs to the incoming blood flow. This vascular reversal provides nourishment to the parasitic twin and, as a consequence, suppresses its cardiac development, leading to acardia or a severely malformed heart. The acardiac twin exhibits a complete reversal of the normal placenta-to-fetus blood flow and, hence, this phenomenon is named twin reversed arterial perfusion sequence [4]. Another theory proposed for the etiology of this anomaly is that of the unequal distribution of cells early on in the pregnancy during monozygotic twining, which could be the reason for the acardia in one of the twins [5].

As a result of the lack of an efficient pumping mechanism in the recipient twin, the lower part of the fetus receives relatively better-oxygenated blood and is thus able to develop more than its cranial end, which receives little or no oxygenation. At this point, the acardiac twin acts like a parasite and has the potential to cause serious consequences in the pump twin such as high output congestive heart failure, eventually leading to hydrops fetalis.

This recipient or parasitic twin can have four different forms of presentation according to the degree of cephalic and truncal maldevelopment [6]:

1) Acardius-acephalus: in which no cephalic end structures are present. The head and upper extremities are lacking. It is the most common presentation. This was the one present in our case.

2) Acardius-anceps: where some cranial structure and neural or brain tissue is present. The body and extremities are also developed. It is the most highly developed form out of all the subtypes.

3) Acardius-acormus: which presents with considerable cephalic end structure; however, truncal structures are not discernable. The umbilical cord is attached to the head end. It is the rarest form of acardia.

4) Acardius amorphous: which is the least developed, and this severe form of malformation makes it unrecognizable as a human fetus. It appears as a completely heterogeneous mass, as neither cranial end structures nor truncal end structures are discernable on imaging. This is differentiated from placental teratoma only by the presence of an umbilical cord attachment.

This reversal of flow in TRAPS can be precisely detected with color Doppler of the umbilical artery. We strongly advocate the use of umbilical artery Doppler scans to rule out the possibility of an acardiac twin when presented with a persistent heterogeneous mass alongside a fetus on serial antenatal ultrasound scans. This, however, was not performed in our case and diagnosis was therefore made postoperatively. Apart from the prognosis of the acardiac twin being lethal, the fetal mortality of the pump twin may be as high as 50% because of high output cardiac failure [7].

Preliminary studies have hypothesized that this mortality may be related to the fraction of blood needed to perfuse the acardiac twin, measured using umbilical venous diameter (UVD) ratios with the help of Doppler imaging. Cases with lower UVD ratios may be more likely to require earlier intervention than otherwise [8]. This can be achieved by in-utero techniques where treatment revolves around the ultrasound-guided or surgical destruction of the inter-twin anastomosis and includes [9-10]:

1) Endoscopic laser coagulation or radio-frequency ablation

2) Surgical (fetoscopic) ligation of acardiac twin umbilical cord

## Conclusions

On the detection of any abnormal mass present alongside a normal fetus during serial antenatal ultrasound scans, the possibility of TRAPS as a differential diagnosis should be considered especially if the presence of two umbilical arteries is detected. Thus, pre-op umbilical artery Doppler imaging should be warranted if the possibility of TRAPS is likely. Furthermore, such anomalies, once discovered, should be followed up judiciously for progression to complications. Such cases should be granted suitable early intervention based on the clinical expertise of the obstetrician and interventional radiologist to salvage the normal twin.

## Figures and Tables

**Figure 4:**
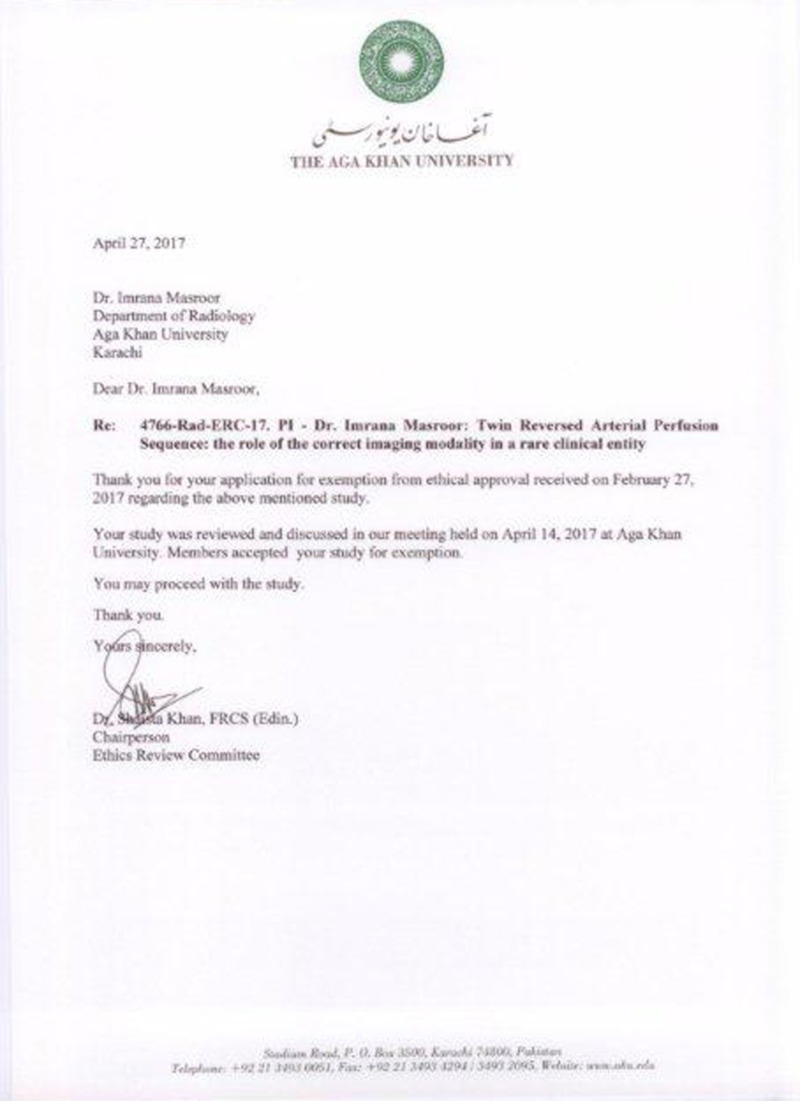
Ethical review committee exemption form
